# Supplementary material for: A dose-finding safety and feasibility study of oral activated charcoal and its effects on the gut microbiota in healthy volunteers not receiving antibiotics
Source: PLoS One. 2022 Jun 14;17(6):e0269986. doi: 10.1371/journal.pone.0269986 (PMC9197061; doi:10.1371/journal.pone.0269986)
Supplement: S1 Appendix — (DOCX) [file pone.0269986.s002.docx]

**Masonic Cancer Center**

**University of Minnesota**

**A pilot study of activated charcoal in healthy volunteers**

**2019LS014**

**IND 143937**

**Principal Investigator:**

Armin Rashidi MD, PhD

Division of Hematology, Oncology and Transplantation

**Co-Investigators:**

Alexander Khoruts, MD

Pamala Jacobson, PharmD

Christopher Staley, PhD*

*non-clinical – will not consent subjects

**Biostatistician:**

Ryan Shanley, MS

**Version Date:**

October 18, 2019

**Confidential**

**Revision History**

| Revision # | Version Date | Summary of Changes | Consent Change |
| --- | --- | --- | --- |
| n/a | 01/22/2019 | Original submission to CPRC | n/a |
|  | 03/29/2019 | Changes based on IRB pre-review:   - Added IND template language - Updated background section (added information on food grade AC, removed references to efficacy) - Updated to clarify that the AC used in this study is food grade - Added volunteer recruitment procedure - Added study withdrawal procedure   Additional Changes:   - Eligibility updated to add Relapsed AML - Clarified study dates for the volunteer stage (drink will be consumed on M/T/W for 3 weeks, not 9 consecutive days) in order to avoid weekend study visits - Minor edits to remove typos | n/a |
|  | 06/25/2019 | Upon advice of the FDA, this study will only test the charcoal in healthy volunteers – study resigned to single stage pilot study; study product will be medical grade AC; additional editsto inclusion/exclustion critier, study drug administration; study monitoring; study calendar as requested by FDA | Yes |
|  | 10/18/2019 | Typographical errors corrected | No |

**IND Sponsor/Principal Investigator Contact Information:**

Armin Rashidi, MD, PhD

Hematology, Oncology and Transplantation

Department of Medicine

420 Delaware Street SE, MMC 480

Minneapolis, MN 55455

612-625-9604 (phone)

612-625-6919 (fax)

arashidi@umn.edu (email)

**Table of Contents**

[Abbreviations 6](#_Toc12367097)

[Protocol Synopsis 7](#_Toc12367098)

[Protocol Schema 8](#_Toc12367099)

[1 Objectives 9](#_Toc12367100)

[1.1 Primary Objective 9](#_Toc12367101)

[1.2 Secondary Objective 9](#_Toc12367102)

[2 Background and Rationale 9](#_Toc12367103)

[2.1 Infectious Complications in Chemotherapy 9](#_Toc12367104)

[2.2 Prevention of Dysbiosis 9](#_Toc12367105)

[2.3 Activated Charcoal 9](#_Toc12367106)

[2.4 Rationale 10](#_Toc12367107)

[3 Study Design 10](#_Toc12367108)

[4 Participant Selection 10](#_Toc12367109)

[4.1 Inclusion Criteria 10](#_Toc12367110)

[4.2 Exclusion Criteria 11](#_Toc12367111)

[5 Patient Registration 11](#_Toc12367112)

[5.1 Participant Identification 11](#_Toc12367113)

[5.2 Study Enrollment and Dose Level Assignment 11](#_Toc12367114)

[5.3 Participants Who Do Not Begin the Study 11](#_Toc12367115)

[6 Study Plan 11](#_Toc12367116)

[6.1 Healthy Volunteers 11](#_Toc12367117)

[6.1.1 Dose Assignment 11](#_Toc12367118)

[6.1.2 Consumption of Study Product (Oral AC) 12](#_Toc12367119)

[6.1.3 Experience Rating 12](#_Toc12367120)

[6.1.4 Compensation 12](#_Toc12367121)

[6.2 Subject Monitoring 13](#_Toc12367122)

[6.3 Management of Selected Expected Toxicities 13](#_Toc12367123)

[6.4 Supportive Care 13](#_Toc12367124)

[6.5 Duration of Study Participation 13](#_Toc12367125)

[6.6 Withdrawal from Study 13](#_Toc12367126)

[7 Schedule of Activities 14](#_Toc12367127)

[8 Study Product Information 14](#_Toc12367128)

[8.1 Medical Grade Activated Charcoal 14](#_Toc12367129)

[8.1.1 Other Names 14](#_Toc12367130)

[8.1.2 Description 14](#_Toc12367131)

[8.1.3 Mode of Action 14](#_Toc12367132)

[8.1.4 Availability 14](#_Toc12367133)

[8.1.5 Storage 15](#_Toc12367134)

[8.1.6 Preparation and Administration 15](#_Toc12367135)

[8.1.7 Toxicities 15](#_Toc12367136)

[9 Event Monitoring, Documentation and Reporting 15](#_Toc12367137)

[9.1 Event Terminology 16](#_Toc12367138)

[9.2 Event Documentation 17](#_Toc12367139)

[9.3 SAE and Death Documentation 17](#_Toc12367140)

[9.4 Expedited Event Reporting 17](#_Toc12367141)

[10 Study Data Collection and Monitoring 18](#_Toc12367142)

[10.1 Data Management 18](#_Toc12367143)

[10.2 Case Report Forms 18](#_Toc12367144)

[10.3 Data and Safety Monitoring Plan (DSMP) 19](#_Toc12367145)

[10.4 Study Monitoring 19](#_Toc12367146)

[10.5 Record Retention 19](#_Toc12367147)

[11 Study Endpoints 20](#_Toc12367148)

[11.1 Primary Endpoint 20](#_Toc12367149)

[11.2 Secondary Endpoint 20](#_Toc12367150)

[12 Statistical Considerations 20](#_Toc12367151)

[12.1 Study Design 20](#_Toc12367152)

[12.2 Sample Size Considerations 20](#_Toc12367153)

[12.3 Statistical Analysis 20](#_Toc12367154)

[13 Ethical and Regulatory Considerations 21](#_Toc12367155)

[13.1 Good Clinical Practice 21](#_Toc12367156)

[13.2 Ethical Considerations 21](#_Toc12367157)

[13.3 Informed Consent 21](#_Toc12367158)

[14 References 21](#_Toc12367159)

[Appendix I - Eligibility Checklist 22](#_Toc12367160)

[Appendix II – Volunteer Product Rating Form 23](#_Toc12367161)

# Abbreviations

| **Abbreviation** | **Definition** |
| --- | --- |
| AC | Activated Charcoal |
| AE | Adverse event |
| AHC | Academic Health Center |
| CDI | Clostridium difficile infection |
| CETI | Cancer Experimental Therapeutics Initiative |
| CFR | Code of Federal Regulations |
| CPRC | Cancer Protocol Review Committee |
| CRF | Case report form |
| CTCAE | Common Toxicity Criteria Adverse Event |
| CTEP | Cancer Therapy Evaluation Program |
| CTO | Clinical Trials Office |
| CTSI | Clinical and translational science institute |
| DSMP | Data and Safety Monitoring Plan |
| IRB | Institutional review board |
| MCC | Masonic Cancer Center |
| MCC-CISS | Masonic cancer center - Clinical Informatics Shares Services |
| NCI | National Cancer Institute |
| ONCORE | Online Enterprise Research Management Environment |
| PHI | Protected Health Information |
| SAE | Serious adverse event |
| SOP | Standard operating procedure |

# Protocol Synopsis

| **Study Design:** | This is a pilot study designed to test feasibility, tolerability, and safety of medical grade oral Activated Charcoal (AC) in 12 healthy volunteers. |
| --- | --- |
| **Primary Objective:** | Compare 2 extemporaneously prepared AC suspensions at 2 doses (4 combinations in total) in healthy volunteers for palatability/tolerability |
| **Patient Population:** | Healthy adult volunteers |
| **Inclusion Criteria:** | - Age >18 years - No use of prescription medications, including oral birth control, currently or in the last 30 days - Sexually active women must be using an effective form of contraception. |
| **Exclusion Criteria:** | - At risk of GI hemorrhage or perforation due to underlying pathology, recent surgery, or medical conditions that could be adversely affected by the administration of AC. - Planning to have an endoscopic procedure - Known hypersensitivity to AC - Non-English speakers |
| **Study Plan:** | In the proposed pilot study, we will compare 2 extemporaneously prepared AC suspensions at 2 doses (4 combinations in total) in healthy volunteers for palatability/tolerability. |
| **Study Product** | Medical Grade Activated Charcoal |
| **Enrollment Plan:** | 12 healthy adults enrolled in a 4 month period |

# Protocol Schema

**
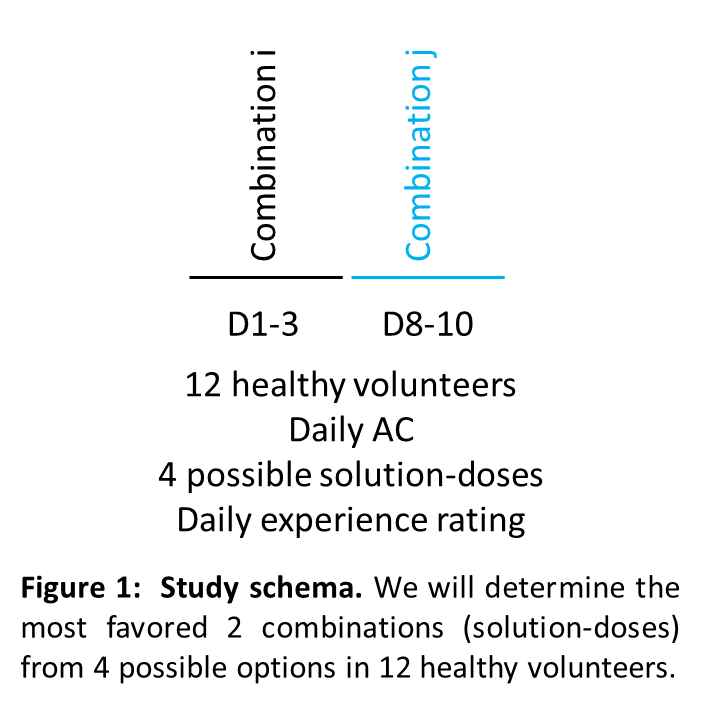
**

# Objectives

## Primary Objective

The primary objective is to compare 2 extemporaneously prepared Medical Grade Activated Charcoal, “AC” suspensions at 2 doses (4 combinations in total) in healthy volunteers for palatability/tolerability.

## Secondary Objective

Determine the incidence of grade 2 and above SAEs deemed at least probably related to AC.

# Background and Rationale

## Infectious Complications in Chemotherapy

Antibiotics disrupt gut microbial communities (dysbiosis). A large fraction of most antibiotics, even when administered intravenously, reaches the colonic lumen and its commensal flora. Antibiotics can reach the gut lumen via mechanisms that include incomplete absorption in the small bowel (e.g., macrolides)^1^, hepatic excretion via bile (e.g., tetracyclines)^2^, or active transfer across the intestinal mucosa into the gut lumen (e.g., fluoroquinolones)^3^.

In the absence of antibiotics, a stable and diverse gut microbiota effectively inhibits colonization by potential pathogens. In the presence of antibiotics, however, pathogenic organisms can grow and cause intestinal and systemic infections. The best example for such infections is *C. difficile* infection (CDI). Even a single dose of an antibiotic (e.g. pre-operative single-dose intravenous antibiotic) can lead to CDI. Surprisingly, no attempts have been made to prevent dysbiosis in individuals exposed to antibiotics. A novel strategy that protects the indigenous microbiota without detracting from the beneficial effects of antibiotics could prevent pathobiont outgrowth and reduce infectious complications of antibiotic use.

## Prevention of Dysbiosis

It is important to discover ways to prevent the untoward consequences of dysbiosis in individuals exposed to antibiotics. The use of oral AC, a potent non-absorbable adsorbent, may protect the gut microbiota by adsorbing the fraction of intravenous (IV) antibiotics that ultimately reaches the intestinal lumen.

## Activated Charcoal

Activated charcoal (AC) is a processed form of carbon with small, low-volume pores that increase the surface area available for adsorption. As a potent adsorbent with no systemic absorption from the gut, AC is a standard and effective agent used in the management of poisonings when gastrointestinal decontamination of drugs or chemicals is indicated.

## Rationale

Investigators are looking for new ways to protect the microbiome during the period of antibiotic exposure, improve the safety of antibiotics and reduce the rate of dysbiosis-related complications in patients. Determining the palatability and tolerability of AC in healthy volunteers in this research will aid in the design of future trials to investigate the efficacy of AC in patients.

# Study Design

We will use the powder form of medical grade oral AC and mix it with liquids for administration. 12 healthy volunteers will be enrolled to determine the two most palatable and tolerable AC combinations. There will be a total of 4 combinations (2 AC doses and 2 solutions) in stage 1. Each participant will drink an assigned combination every day for 3 consecutive days (Monday, Tuesday, Wednesday, “M/T/W”) and switch to a different assigned combination M/T/W the following week. AC solution assignments will be defined before the study using a balanced incomplete block design. Each subject will rate their experience using a 5-point scale every day for the 12 days they are on study.

The following outcome variables will be measured:

1. Each healthy volunteer will rate their experience using a 5-point scale (1=terrible, 2=bad, 3=okay, 4=good, 5=great) every day.
2. To evaluate tolerability, after each round of study product consumption, a member of the study team will contact the subject and screen for grade 2 or higher AEs and SAEs

# Participant Selection

Study entry is open to adult volunteers regardless of gender or ethnic background. While there will be every effort to seek out and include women and minorities, the subject population is expected to reflect the population of Minnesota.

## Inclusion Criteria

- - - - Age ≥18 years
      - No use of prescription medications, including oral birth control, currently or in the last 30 days
      - Sexually active women must be using an effective form of contraception.
      - Voluntary written consent signed before performance of any study-related procedure

## Exclusion Criteria

- - - - At risk of GI hemorrhage or perforation due to underlying pathology, recent surgery, or medical conditions that could be adversely affected by the administration of AC.
      - Planning to have an endoscopic procedure
      - Known hypersensitivity to AC
      - Non-English speakers

# Patient Registration

Registration will occur after the participant has signed the subject consent and eligibility is confirmed, but before any study procedures have occurred. To be eligible for registration to this study, the participant must meet each criteria listed on the eligibility checklist (found in appendix I) based on the eligibility assessment documented in the participant’s research record.

## Participant Identification

Healthy Volunteers will respond to poster advertisements containing a brief study description and the contact information of the study coordinator.

## Study Enrollment and Dose Level Assignment

Upon completion of the screening evaluation, eligibility confirmation and obtaining written consent the Study Coordinator or designee will enroll the volunteer on study in OnCore.

The Primary Clinical Research Coordinator (PCRC) or designee will assign the study dose-solution and add the on-study date to complete enrollment.

## Participants Who Do Not Begin the Study

If a participant is registered to the study, and is later found not able to begin the study, for any reason, the participant will be taken off study. The Study Coordinator or designee will update OnCore of the participant’s non-study status. The participant will be replaced to fulfill enrollment requirements.

# Study Plan

## Healthy Volunteers

### Dose Assignment

|  | Days 1-3  (M,T, W – week 1) | | Days 8-10  (M,T, W – week 2) | |
| --- | --- | --- | --- | --- |
| Subject | Solution | Dose | Solution | Dose |
| 1 | A | 1 | B | 2 |
| 2 | A | 2 | B | 1 |
| 3 | B | 1 | A | 2 |
| 4 | B | 2 | A | 1 |
| 5 | A | 1 | B | 2 |
| 6 | A | 2 | B | 1 |
| 7 | B | 1 | A | 2 |
| 8 | B | 2 | A | 1 |
| 9 | A | 1 | B | 2 |
| 10 | A | 2 | B | 1 |
| 11 | B | 1 | A | 2 |
| 12 | B | 2 | A | 1 |

Dose assignments will be defined at the time of study registration using a balanced incomplete block design (Table 1).

The dose levels are 12 and 25 of medical grade oral AC. While the standard AC dose for poison control is 25-100 grams, these smaller doses have been chosen to ensure safety. The AC will be mixed with at least 4 oz of either tap water or apple juice for a total of 4 combinations.

### Consumption of Study Product (Oral AC)

Participants will report to the University of Minnesota Health Clinical Research Unit (CRU) on a daily basis and consume the assigned combination of oral AC. Each participant will drink an assigned combination every day for 3 consecutive days (M/T/W) and switch to a different assigned combination for the 3 days (M/T/W) the following week. Subjects will drink AC in sitting position. Subjects must stay in the research unit for 15 minutes after consuming AC to be observed for aspiration.

### Experience Rating

After drinking the solution, each subject will rate their experience using a 5-point scale (1=terrible, 2=bad, 3=okay, 4=good, 5=great) every day (Appendix III). They will be asked to do this daily starting day 1 (Mon of week 1) through day 12 (Friday of week 2).

### Compensation

Each of the healthy volunteers participating this pilot study will receive an Amazon gift card for $100.

## Subject Monitoring

Healthy volunteers will be observed for fifteen minutes after consuming the AC. Upon leaving the research unit, they will have access to the CRA via phone. For 2 days after each round of study product consumption, a member of the study team will contact the subject and screen for all grade 2 or higher AEs and SAEs.

Subjects with an adverse event will be followed until the event has resolved or stabilized with sequelae.

## Management of Selected Expected Toxicities

Toxicity data is based on studies using pharmaceutical grade AC for treatment of intentional overdose/poison control. These doses are much higher than the planned doses of medical grade AC on this protocol. Lower doses are thought to be more tolerable.

- The most common toxicity of AC (doses used for poisoning treatment) is nausea/vomiting, ranging from 15-27%.
- Pulmonary aspiration is the most serious complication, occurring in <1% of patients. AC will not be administered to patients at risk for aspiration.

It should be noted that the cause of aspiration is multifactorial, and part of the risk reported in studies should be contributed to the effect of toxin or the loss of protective reflexes in cases of poisoning. We do not expect these to be present in our participants.

## Supportive Care

No specific supportive care is needed.

## Duration of Study Participation

Participants will be involved for 12 days over a 2 week period including 6 days of active study participation and 4 days of study team follow-up.

## Withdrawal from Study

Participants have the right to withdraw consent at any time by informing a member of the study staff or by following the instructions provided in the HIPAA document.

# Schedule of Activities

|  | Screening Visit | Week 1-2 |
| --- | --- | --- |
| Consent and eligibility screening | X |  |
| Physical Exam | X |  |
| Drink Study product in either tap water, protein shake, or apple juice (per table 1*) |  | M, T, W |
| Rate Experience (taste test) |  | M, T, W |
| Screen for AEs |  | M, T, W |
| Telephone call from study staff to check for AEs |  | Th, F |

# Study Product Information

## Medical Grade Activated Charcoal

### Other Names

Medical grade AC, “Activated Charcoal Powder, USP”, activated carbon.

### Description

Activated Charcoal, Powder, USP is carbon that has been treated to create low-volume pores that increase the area available for chemical reactions and adsorption. The most common pharmaceutical uses of activated charcoal is as a purification agent and antitoxin. All Spectrum Chemical USP products are manufactured, packaged and stored under current Good Manufacturing Practices (cGMP) per 21CFR part 211 in FDA registered and inspected facilities.

### Mode of Action

Hemoperfusion through columns of activated charcoal to remove endogenous or exogenous toxins in uremia, hepatic failure, or acute toxicity associated with overdose of certain drugs.

### Availability

Medical grade AC, “Activated Charcoal Powder, USP” will be purchased from Spectrum Chemical MFG Corporation. https://www.spectrumchemical.com Spectrum is an FDA registered manufacturer and distributor of chemicals and laboratory products.

### Storage

As per the Spectrum Safety Data Sheet, the container of AC should be kept tightly closed in a dry and well-ventilated place. The container should be stored at room temperature.

### Preparation and Administration

A member of the study team will make a suspension using 12g or 25g, or 50g of AC mixed with at least 4 oz. of either tap water or apple juice. The solutions will be administered as per section

### Toxicities

**GI Effects**

May cause vomiting, constipation, diarrhea, and GI obstruction or fecal impaction in dehydrated patients.

Generally should not be used when decreased peristalsis present (reduced or absent bowel sounds); if risk of GI obstruction, perforation, or hemorrhage exists; if surgery has occurred recently; or if electrolyte imbalance or volume depletion exists.

**Pulmonary Effects**

Aspiration of activated charcoal may lead to more severe complications than aspiration of gastric contents alone. Aspiration from vomiting or misdirected nasogastric catheter has resulted in granulomatous reactions, bronchiolitis obliterans, tissue reaction to suspension agents (sorbitol, povidone), increased lung permeability, and rarely, death.

**Use of Fixed Combination**

When used in fixed combination with other agents, consider the cautions, precautions, and contraindications associated with the concomitant agents.

**Common Adverse Effects**

Vomiting, diarrhea, constipation, black stools.

# Event Monitoring, Documentation and Reporting

Toxicity and adverse events will be classified and graded according to NCI's Common Terminology Criteria for Adverse Events version 5.0 (CTCAE V5) and reported on the schedule below. A copy of the CTCAE can be downloaded from the CTEP home page. (<http://ctep.cancer.gov/protocolDevelopment/electronic_applications/ctc.htm#ctc_50>).

The following definitions of adverse events (AEs) and serious adverse events (SAEs) will determine whether the event requires expedited reporting via the SAE Report Form in addition to routine documentation in the OnCore AE case report form (CRF).

Note: throughout this section the generic term “study drug” refers to study product, medical grade Oral AC.

## Event Terminology

Adverse Event: Any untoward medical occurrence associated with the use of a drug in humans, whether or not considered drug related.

Serious Adverse Event: An adverse event is considered “serious” if, in the view of either the investigator or sponsor, it results in any of the following outcomes:

- Death
- A life-threatening adverse experience
- Inpatient hospitalization or prolongation of existing hospitalization
- A persistent or significant incapacity or substantial disruption of the ability to conduct normal life functions
- A congenital anomaly/birth defect
- Important medical events that may not result in death, be life-threatening, or require hospitalization may be considered serious when, based upon appropriate medical judgment, they may jeopardize the patient or subject and may require medical or surgical intervention to prevent one of the outcomes listed in this definition.

Unexpected Event: An adverse event or suspected adverse reaction is considered “unexpected” if it is not listed in protocol-related documents (e.g. protocol, consent documents, investigator brochure, package insert), or is not listed at the specificity or severity that has been observed or given the characteristics of the subject population being studied.

Attribution: is the relationship between an adverse event or serious adverse event and the study drug. Attribution is assigned as follows:

- Definite – The AE is clearly related to the study drug
- Probable – The AE is likely related to the study drug
- Possible – The AE may be related to the study drug
- Unlikely – The AE is doubtfully related to the study drug
- Unrelated – The AE is clearly not related to the study drug

Attribution must be assigned by the treating physician or the PI.

The following definitions are from the Masonic Cancer Center’s Standard Operating Procedure (SOP) Deviation Reporting:

Major Deviation: A deviation or violation that impacts the risks and benefits of the research; may impact subject safety, affect the integrity of research data and/or affect a subject’s willingness to participate in the research. Deviations that place a subject at risk, but do not result in harm are considered to be major deviations.

Minor Deviation: A deviation or violation that does not impact subject safety, compromise the integrity of research data and/or affect a subject’s willingness to participate in the research.

## Event Documentation

Participants will be monitored for adverse events from the 1^st^ dose of study treatment through day 12. After that time point, events meeting the definition of serious and at least possibly related to study treatment will be documented upon knowledge.

We will collect the following Grade 2-5 adverse events attributable to AC:

- Aspiration of activated charcoal

In addition any event meeting the definition of a serious adverse event (SAE) regardless of attribution that occurs during this period will be documented in the source document and recorded in OnCore.

## SAE and Death Documentation

Any event meeting the definition of a serious adverse event (SAE) requires documentation using the MCC SAE Report Form in OnCore.

## Expedited Event Reporting

| **Agency reporting to** | **Criteria for reporting** | **Timeframe** | **Form to Use** | **Submission address/email address** |
| --- | --- | --- | --- | --- |
| **U of MN IRB** | Unanticipated death of a locally enrolled subject(s); New or increased risk; Any adverse event that require a change to the protocol or consent form – refer to the IRB website for complete details | 5 Business Days | IRB Report Form | irb@umn.edu |
|  | Clinical deviations per current IRB reporting requirements | | OnCore Deviation Form |  |
| **Masonic Cancer Center**  **SAE Coordinator** | Events that meet the definition of dose limiting toxicity or an early study stopping rule | At time of reporting | Event Form | mccsaes@umn.edu |
| **FDA** | Unexpected and fatal or unexpected and life threatening suspected adverse reaction | no later than 7 Calendar Days | MCC SAE Report Form | Submit to FDA as an amendment to IND |
|  | 1) Serious and unexpected suspected adverse reaction or  2) increased occurrence of serious suspected adverse reactions over that listed in the protocol or investigator  brochure or  3) findings from other sources (other studies, animal or in vitro testing) | no later than 15 Calendar-Days |  |  |

# Study Data Collection and Monitoring

## Data Management

This study will collect regulatory and clinical data using University of Minnesota CTSI’s instance of OnCore® (Online Enterprise Research Management Environment). The Oncore database resides on dedicated secure and PHI compliant servers. All relevant AHC IS procedures related for PHI compliant servers (as required by the Center of Excellence for HIPAA Data) apply to Oncore databases.

The data will be integrated and extracted to researchers through the CTSI Informatics team and will be delivered through secure and compliant mechanisms (e.g. AHC IE data shelter, BOX, sftp, etc). If data de-identification is needed, then compliant AHC IE data de-identification tools will be used. The informatics team will grant the IRB approved study team members access to data.

Key study personnel are trained on the use of OnCore and will comply with protocol specific instructions embedded within the OnCore.

## Case Report Forms

Participant data will be collected using protocol specific electronic case report forms (e-CRF) developed within OnCore based on its library of standardized forms. The e-CRF will be approved by the study’s Principal Investigator and the Biostatistician prior to release for use. The Primary Clinical Research Coordinator or designee will be responsible for registering the patient into OnCore at time of study entry, completing e-CRF based on the patient specific calendar, and updating the patient record until patient death or end of required study participation.

## Data and Safety Monitoring Plan (DSMP)

The study’s Data and Safety Monitoring Plan will be in compliance with the University of Minnesota Masonic Cancer Center's Data & Safety Monitoring Plan (DSMP), which can be accessed at http://z.umn.edu/dmsp.

For the purposes of data and safety monitoring, this study is classified as high risk. Therefore the following requirements will be fulfilled:

- - - At least quarterly review of the study’s progress by the Masonic Cancer Center Data and Safety Monitoring Council (DSMC).
    - The PI will comply with at least twice yearly monitoring of the project by the Masonic Cancer Center monitoring services.
    - The PI will oversee the submission of all reportable adverse events per section 9.3 to the Masonic Cancer Center’s SAE Coordinator, the University of Minnesota IRB, and the FDA.

In addition, at the time of the continuing review with the University of Minnesota IRB, a copy of the report with any attachments will be submitted to the Cancer Protocol Review Committee (CPRC).

**IND Annual Reports**

In accordance with regulation 21 CFR § 312.33, the Sponsor-Investigator will submit a progress report annually. The report will be submitted within 60 days of the anniversary date that the IND went into effect.

## Study Monitoring

The investigator will permit study-related monitoring, audits, and inspections by the study’s Principal Investigator and/or any designees, the local IRB, government regulatory bodies, and University of Minnesota compliance groups. The investigator will make available all study related documents (e.g. source documents, regulatory documents, data collection instruments, study data, etc.). The investigator will ensure the capability for inspections of applicable study-related facilities (e.g. pharmacy, diagnostic laboratory, etc.) will be available for trial related monitoring, audits, or regulatory inspections.

## Record Retention

The investigator will retain study records including source data, copies of case report form, consent forms, HIPAA authorizations, and all study correspondence in a secured facility for at least 6 years after the study file is closed with the IRB and FDA.

# Study Endpoints

## Primary Endpoint

The primary endpoint is the average palatability rating (on a 1-5 scale) of each treatment combination.

## Secondary Endpoint

Safety, defined by the incidence of grade 2 and above AEs deemed at least probably related to AC by day 12.

# Statistical Considerations

## Study Design

This is a pilot study designed to test tolerability and safety of oral AC in 12 healthy volunteers.

We will follow a predetermined treatment assignment using the balanced incomplete block design shown in Section 6.1.1. Twelve healthy subjects will be assigned two dose and solution combinations to take for three days each. Each subject will receive each dose once and each solution once. Different subjects will receive different combinations or different sequences. Each of the four combinations will be given to six subjects total, allowing straightforward comparisons. Each solution (or dose) precedes each of the others for exactly half of the subjects, to minimize confounding due to the ordering of the treatment assignments.

## Sample Size Considerations

The sample size was chosen so that each combination could be given the same number of times.

## Statistical Analysis

We will average each subject’s score over the 3 days of each treatment. If a subject misses one or more days, we will impute the average of the known days for the unknown days. We will also record the reported reason for not taking AC, and based on that we may adjust the imputation procedure on a case-by-case basis. Since the goal is to pick the most tolerable/palatable combination, we will choose the one with the highest average score; no statistical hypothesis tests are required. We will also consider the combination with the fewest percentage of “terrible” or “bad” ratings, although this is expected to be associated with average score.

# Ethical and Regulatory Considerations

## Good Clinical Practice

The study will be conducted in accordance with the appropriate regulatory requirement(s). Essential clinical documents will be maintained to demonstrate the validity of the study and the integrity of the data collected. Master files should be established at the beginning of the study, maintained for the duration of the study and retained according to the appropriate regulations.

## Ethical Considerations

The study will be conducted in accordance with ethical principles founded in the Declaration of Helsinki. The IRB will review all appropriate study documentation in order to safeguard the rights, safety and well-being of the patients. The study will only be conducted at sites where IRB approval has been obtained. The protocol, informed consent, written information given to the patients, safety updates, annual progress reports, and any revisions to these documents will be provided to the IRB by the investigator.

## Informed Consent

All potential study participants will be given a copy of the IRB-approved Consent to review. The investigator or designee will explain all aspects of the study in lay language and answer all questions regarding the study. If the participant decides to participate in the study, he/she will be asked to sign and date the Consent document. Patients who refuse to participate or who withdraw from the study will be treated without prejudice.

# References

1. Nord, C. E. & Edlund, C. Impact of antimicrobial agents on human intestinal microflora. *J. Chemother.* **2,** 218–37 (1990).

2. Agwuh, K. N. & MacGowan, A. Pharmacokinetics and pharmacodynamics of the tetracyclines including glycylcyclines. *J. Antimicrob. Chemother.* **58,** 256–265 (2006).

3. Lowes, S. & Simmons, N. L. Multiple pathways for fluoroquinolone secretion by human intestinal epithelial (Caco-2) cells. *Br. J. Pharmacol.* **135,** 1263–75 (2002).

# Appendix I - Eligibility Checklist

**A pilot study of activated charcoal in healthy volunteers**

**Eligibility Checklist – page 1 of 1**

Participant ID 19014-UMN-____ (assigned in OnCore)

**INCLUSION CRITERIA**

**A “NO” response to any of the following disqualifies the participant from study entry.**

|  |  | **Yes No** |
| --- | --- | --- |
| 1. **1** | Age ≥18 years |  |
| 1. **2** | No use of prescription medications, including oral birth control, currently or in the last 30 days |  |
| 1. **3** | Sexually active women must be using an effective form of contraception. |  |
| 1. **4** | Voluntary written consent signed before performance of any study-related procedure |  |

**EXCLUSION CRITERION**

**A “YES” response to the following disqualifies the participant from study entry.**

|  |  | **Yes No** |
| --- | --- | --- |
| **1** | At risk of GI hemorrhage or perforation due to underlying pathology, recent surgery, or medical conditions that could be adversely affected by the administration of AC. |  |
|  | Planning to have an endoscopic procedure |  |
|  | Known hypersensitivity to AC |  |
|  | Non-English speakers |  |

Date consent form signed:

Having obtained consent and reviewed each of the inclusion/exclusion criteria, I verify that this participant is eligible.

Signature of person verifying eligibility Date

# Appendix II – Volunteer Product Rating Form

Study ID: _____________________________

Date: ________________________

1. Please rate your charcoal experience today by choosing one the 5 items below


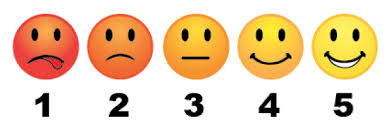


1. Have you had any issues since last drink of charcoal that you think may be related to your charcoal experience?

______________________________________________________________

______________________________________________________________

______________________________________________________________
