# Supplementary material for: A dose-finding safety and feasibility study of oral activated charcoal and its effects on the gut microbiota in healthy volunteers not receiving antibiotics
Source: PLoS One. 2022 Jun 14;17(6):e0269986. doi: 10.1371/journal.pone.0269986 (PMC9197061; doi:10.1371/journal.pone.0269986)
Supplement: Attachment — Submitted filename: Response to Reviewers.docx [file pone.0269986.s003.docx]

**We thank the reviewers for their constructive feedback. Changes in the text are highlighted in yellow.**

**Reviewer #1**

**Comment:** Indicate the date range subjects were enrolled in the study.

**Response:** Enrollment occurred in Nov 2020. This was added to the revised manuscript.

**Comment:** Page 6: In addition to stating the frequencies of AEs, provide the corresponding percentages.

**Response:** This information was added to the revised manuscript.

**Comment:** Indicate if AEs were collected according to standard methods, i.e. CTCAE.

**Response:** AEs were collected according to CTCAE v.5.0. This is highlighted in the revised manuscript.

**Reviewer #2**

**Comment:** This study nicely shows that OAC can be made palatable and does not appear to adversely affect the microbiome when dosed intermittently at low levels. I agree that the low dose OAC with apple juice was liked by the most patients, but medicine doesn’t always have to taste nice and an equally important question to be answered later is what is the correct dose for preventing antibiotic microbiome effects? It could be 12g, it could be 25g, it could be more. What they actually showed is that lower dose OAC is more palatable and mixed with apple juice is more palatable. This should be described in the results and acknowledged in the discussion. They also subsequently say the most likely scenario for this treatment's use would be in surgical patients on IV antibiotics, where presumably the OAC therapy would only be needed 1-3 times at most. Maybe not so big a deal how nice it tastes.

**Response:** We agree. To conduct the future trial in surgical patients, we needed a pilot study to get some idea about an appropriate dose/solution, understanding that it might not be the best choice. This information did not exist before this pilot study. Factors that could influence the optimal choice included palatability, potential side effects, feasibility, and compliance. However, as the reviewer correctly pointed out, a therapeutic effect may occur at various doses (not just the one we found as preferred among the tested options) and palatability of the solution may not be critical when consumed only once or a few times. We have added these to the revised manuscript (Introduction and Discussion) and changed “the preferred” to “a suitable” in the beginning of Discussion.

**Comment:** The microbiome data is nice and very clearly shows that within an individual the OAC made no great difference. But this was in 12 people minus the dropout. Not really enough to say conclusively that OAC does not exert its anti-bloating effects by altering the microbiome.

**Response:** This is a valid point. All we can say is that this study did not identify an effect on the microbiome. We have adjusted the language in the revision.

**Reviewer #3**

This is an interesting manuscript describing the dose and feasibility of OAC on the gut microbiota. It is not really an RCT given that only one arm is present and only before and after samples are used and compared within individuals. It is however, important information before the start of RCTs with antibiotics and OAC. I only have a few comments.

**Comment:** Consort diagram: would it be possible to add to the consort diagram the breakdown of people receiving the different amounts and the loss to follow-up for each of these?

**Response:** This information was added to the consent diagram.

**Comment:** It would be good to have a table with some of the characteristics for the participants e.g. gender, age, BMI. This could also be added as text.

**Response:** Unfortunately, we did not collect age and BMI. Gender was added to the revised manuscript in the text.

**Comment:** The methods seem to suggest that the gut microbiota samples after were obtained prior to dose 4, the results suggest that the results were obtained after the two weeks (6 doses). Please check and make consistent.

**Response:** Thank you for this clarifying question. Samples were indeed collected at baseline and after the first week (block 1), prior to dose 4. The study was designed like this because each subject went through 2 blocks, each with a different dose and solution. We would have not been able to attribute the information obtained from post-week 2 samples to week 1 vs. week 2 preparation, so we did not collect an end-of-study sample. The reason subjects went through block 2 was clinical (to assess AEs and tolerability). In the revised manuscript, we added a sentence in the beginning of Statistical Analysis which clarifies that our notation “post-OAC” refers to the pre-dose 4 sample.

**Comment:** Was the increased palatability of the OAC in apple juice due to masking of the taste? Would it therefore be possible to dissolve the OAC in other types of juices or other flavoured drinks as well?

**Response:** OAC does not have any taste, but creates a somewhat uncomfortable sensation in the mouth/throat which was mitigated by dissolving it in the juice. We did not test other types of juice, but it is very possible that they would have the same effect as apple juice. We added this possibility in the revised manuscript (Conclusions, 1st paragraph).

**Comment:** How many doses do the authors think people should take when they receive IV prophylactic antibiotics in conjunction with procedures in order to protect the gut microbiota?

**Response:** This is a very good question that only a trial in patients would be able to answer with certainty. We speculate that 1 dose would be enough but this definitely needs to be tested in a trial. To avoid conveying a premature message to the readers, we did not make a speculation in the manuscript. In the last paragraph of the revised manuscript, we briefly discuss this.
